# Synergistic effects of high-temperature curing and elemental conditioning on red mud-based geopolymer: Compressive strength and immobilization

**DOI:** 10.1371/journal.pone.0343975

**Published:** 2026-04-20

**Authors:** Dongliang Luo, Yufeng Du, Jintao Zhang, Feiyong Chen, Yang Song

**Affiliations:** Institute of Resources and Environment Innovation, Shandong Jianzhu University, Shandong Jinan, China; SASTRA Deemed University, INDIA

## Abstract

Red mud, a by-product of alumina refining, is accumulating rapidly and harming ecosystems. This study converted red mud and fly ash into a geopolymer (RFGP) to create value while mitigating waste. The effects of different sodium silicate moduli and red mud contents on the compressive strength and microstructure (as characterized by SEM, XRD, TG, and FTIR) of RFGP cured for 28 days were systematically analyzed. The results showed that the compressive strength of RFGP decreased with the increase of sodium silicate modulus and red mud content, with minimum 28-day values of 9.65 MPa and 12.81 MPa, respectively. It was found that the percentages of SiO_2_, Al_2_O_3_, and CaO in the raw materials were directly proportional to the compressive strength, while Na_2_O and Fe_2_O_3_ were inversely proportional thereto. Therefore, the chemical composition of substances in the raw material had an important influence on the compressive strength. In addition, the environmental safety of RFGP was evaluated. After the polymer reaction, the heavy metal ions in RFGP were transformed into more stable forms, with the concentration of Hg being significantly reduced, meeting the requirements of applicable environmental standards. This synergistic utilization of industrial by-products demonstrated a viable pathway for the sustainable valorization of hazardous wastes as value-added construction materials.

## 1. Introduction

Red mud is a major by-product of bauxite refining; its properties and characteristics vary with the production route and the quality of the bauxite [1]. In general, the Bayer process generates roughly 1.5 to 2.5 tonnes of red mud per tonne of alumina produced [2]. In recent years, as global alumina production has increased steadily, red mud production has also increased gradually. In 2024, China produced 85 million tonnes of alumina and 107 million tonnes of red mud, yet the utilization rate was only 9.8% [3], leaving vast quantities of unused red mud stockpiled [4]. Most of this material is ultimately disposed of by damming and long-term stockpiling [5]. Red mud slurry (15% to 40% solids) has a pH of 10 to 13.5, a particle size of 0.005 to 0.074 mm, and a Na_2_O content of 2% to 16%. The principal constituents of red mud are CaO, SiO_2_, Al_2_O_3_, Fe_2_O_3_, TiO_2_, and Na_2_O [6], together with trace elements (Y, Zr, Ga, Sc, V), heavy metals (Cr, Mn, Ni, Pb) and occasional elements (U, Th) [7]. Because these minor components are extremely difficult to recover, the mud becomes an environmental liability. The accumulation of red mud not only occupies a large amount of land resources but also may pollute soil and groundwater through percolation [8], posing a long-term threat to the ecological environment. Consequently, developing efficient, environmentally sound treatment and resource-recovery strategies for red mud is essential for sustainable development.

Against the backdrop of growing resource constraints and environmental problems, identifying efficient and eco-friendly approaches to waste resource utilization has emerged as a critical focus in scientific research and engineering practice. As a novel inorganic polymer material, geopolymer, with its excellent physical and mechanical properties, durability, and environmental friendliness [9], shows broad application prospects in the fields of civil engineering, environmental engineering, and geological engineering. It offers a new approach to waste resource utilization by converting industrial by-products or natural minerals into high-strength, durable cementitious materials through specific chemical reactions. Owing to its unique chemical composition (rich in aluminosilicates) and physical structure, red mud exhibits performance deficiencies when used in conventional building materials such as Portland cement. However, these very characteristics are essential and advantageous in geopolymer materials [10]. Thus, red mud represents an ideal raw material for geopolymer synthesis. Through controlled activation and reaction conditions, aluminosilicates in red mud can react with alkali activators. The formation of three-dimensional network structures significantly enhances the physical and mechanical properties of the resulting geopolymers. Moreover, the research, development, and application of red mud-based geopolymers not only expand the scope of red mud utilization but also contribute to the immobilization and encapsulation of heavy metal ions, radioactive elements, and other harmful components in red mud [11]. Consequently, this technology holds promising development prospects and opens up a new pathway for the high-value utilization of red mud.

Current studies have demonstrated the properties of red mud-based geopolymers. The multi-component geopolymer incorporating red mud exhibits improved performance and environmental benefits. The synergistic effect of different solid waste combinations has great potential for recycling solid waste and reducing environmental burdens [12]. Hu et al. [13] prepared red mud-based geopolymers using sodium hydroxide as an alkaline activator. When cured at 20°C for 7 days, the resulting geopolymer exhibited a compressive strength of 6.5 MPa. Hao et al. [14] prepared geopolymers with 70% red mud and 30% fly ash. Their 7-day compressive strength reached 7.5 MPa. Microstructural and compositional analyses indicated that the final product primarily consisted of an amorphous geopolymer binder filled with crystalline phases [15]. Building on these findings, the present study introduces a high-temperature curing process, where the temperature is raised to 60°C while the humidity is maintained at 90 ± 5%. This is because high temperature and sufficient moisture promote the formation of amorphous geopolymer [16]. This is conducive to the improvement of the compressive strength of the RFGP. Bai et al. [17] obtained high-strength red mud-based geopolymers by optimizing the alkaline activator. The geopolymer formulated with red mud and fly ash as precualumina ratio, their research mainly focused on the macroscopic performance characterization. Existing research is limited in analyzing the specific impact of factors such as the silica-alumina ratio. Therefore, to further investigate the impact of elemental ratios on the performance and mechanism of RFGP, this study adjusted the elemental ratios by altering the proportions of RM and FA in the precursors. Strength variations among materials with different elemental ratios were compared to examine the directional effects of these ratios on gel structure formation. Thus, the impact of elemental ratios on the performance of RFGP was determined.

This study successfully developed a high-strength geopolymer from red mud (RM) and fly ash (FA), which are rich in active substances, without altering the inherent characteristics of the raw materials. The research particularly highlighted the critical roles of curing temperature and elemental ratios. RM and FA were used as precursors and sodium silicate as an activator; the performance and mechanism of the prepared RFGP were analyzed through macroscopic (compressive strength) and microscopic (X-ray diffraction, Fourier transform infrared spectroscopy, thermogravimetric analysis, scanning electron microscopy, among others) testing methods. The findings of this work contribute to the expansion and enrichment of utilization pathways for red mud and fly ash and the promotion of their resource utilization.

## 2. Materials and methods

### 2.1. Materials

The red mud used in the study was Bayer process red mud from an aluminum plant in XINFA GROUP. The raw material appeared as brownish-red lumps. It was dried in an oven at 105°C for 24 hours and then crushed and sieved through a 100-mesh screen. The fly ash was supplied by a power plant of Shenhua Technology Development Co., Ltd.; it appeared as a gray, powdery material. The physical and chemical properties of the raw materials were characterized using X-ray fluorescence spectroscopy (XRF) and X-ray diffraction (XRD). As can be seen from Table 1, the main components in red mud include Al_2_O_3_, Fe_2_O_3_, SiO_2_, Na_2_O, TiO_2_, and CaO. With an Fe_2_O_3_ content of about 30% and a CaO content of about 1%, the red mud is identified as typical Bayer red mud, characterized primarily by its high iron and low calcium content. The CaO content of fly ash is only 4.45 wt%, indicating that it belongs to Class F low-calcium fly ash [18], with high contents of Al_2_O_3_ and SiO_2_. As can be seen from S1 Fig in the Appendix, the main mineral phases in fly ash are quartz, calcium hydroxide, hematite, calcium oxide, and among others. The main mineral phases in red mud are calcium oxide, hematite, and others [19]. There is no broad peak representing amorphous material in red mud, indicating that all the aluminosilicates in red mud are present in crystalline form.

**Table 1:** Main chemical composition of raw materials (%).

	Al_2_O_3_	Fe_2_O_3_	SiO_2_	Na_2_O	TiO_2_	CaO	Others
Red mud	34.14	31.81	15.72	10.29	5.44	1.03	1.57
Fly ash	36.22	4.48	47.05	1.22	1.26	4.45	5.32

The alkaline activator used in this study was liquid sodium silicate obtained from Shandong Yousuo Chemical Technology Co., Ltd. Its modulus is 3.30, and its solid content is 35.5 wt% (26.5 wt% SiO_2_, 8.3 wt% Na_2_O). Analytical grade granular sodium hydroxide (purity 96%) was used to adjust the modulus (SiO_2_/Na_2_O molar ratio) of the sodium silicate. The water used was laboratory-prepared ultrapure water.

### 2.2. Experimental method

**Fig 1:**
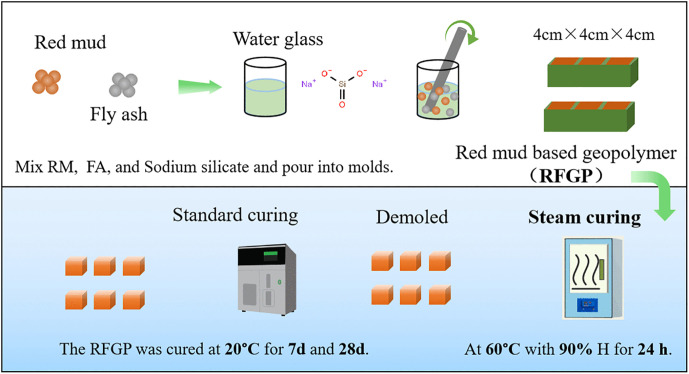
Flowchart of the experimental procedures.

The RFGP preparation process is illustrated in Fig 1. Sodium hydroxide was first mixed with a sodium silicate solution to prepare mixed solutions of different moduli. The mixed solutions were allowed to stand for 24 h after preparation, to dissipate the heat generated during mixing as well as to prevent coagulation. The treated red mud, fly ash, and alkaline activator solutions were accurately weighed with an electronic balance according to the experimental mix proportions shown in Table 2. In this experiment, the liquid-to-solid ratio was fixed at 0.42. The raw materials and alkali activator were mixed thoroughly. The mixture was then poured into a mold coated with a release agent, compacted, and vibrated to remove entrapped air. The specimens were placed in a curing chamber maintained at 60°C and 90% relative humidity for 24 h and then demolded. Afterward, they were placed in a standard curing chamber maintained at 20 ± 2°C and 90 ± 5% relative humidity [20]. The properties of the specimens were tested after curing periods of 7 and 28 days.

**Table 2:** Different Red Mud Content.

Serial number	Sample number	Red mud	Fly ash	sodium silicate modulus
1	R0F100	0%	100%	1.2
2	R10F90	10%	90%	1.2
3	R30F70	30%	70%	1.2
4	R50F50	50%	50%	1.2
5	R70F30	70%	30%	1.2
6	R90F10	90%	10%	1.2
7	R100F0	100%	0%	1.2
8	R30F70-1.0	30%	70%	1.0
9	R30F70-1.2	30%	70%	1.2
10	R30F70-1.4	30%	70%	1.4
11	R30F70-1.6	30%	70%	1.6
12	R30F70-1.8	30%	70%	1.8
13	R30F70-2.0	30%	70%	2.0

### 2.3. Measurements and analysis

#### 2.3.1. Characterization tests.

To terminate further reaction, samples of the appropriate age were ground and sieved through a 200-mesh sieve. The collected powder was then soaked in anhydrous ethanol for 24 hours and dried in an oven at 60°C for 24 hours before removal.

After being poured into the 40 × 40 × 40 mm mold, the mixture was sealed with a plastic film. The specimens were demolded after one day of curing in a constant temperature oven at a set temperature and humidity. Following demolding, the specimens were cured at room temperature to the design age for testing. The compressive strength test was carried out on a TYE-300 pressure tester, with the loading rate set at 2.4 kN/s. RFGP specimens were selected at ages of 7 and 28 days for compressive strength tests. Three cubes were tested per group, and the arithmetic mean was calculated.

Samples were analyzed by scanning electron microscopy (SEM) using a MERLIN Compact scanning electron microscope from Zeiss, Germany. Thermogravimetric analysis (TG-DTG) was performed using a TA Q5000 model instrument (USA). The testing conditions involved heating to 800°C under nitrogen protection at a rate of 20°C/min. KBr and raw material powder were mixed and pressed, and the functional group changes of the samples with different ages and ratios were analyzed by Fourier Transform Infrared Spectroscopy (FTIR). FTIR spectra were acquired using an FTIR-60 spectrometer, over the range of 4000–400 cm^-1^. The XRD tests were carried out using a Rigaku SmartLab X-ray powder diffractometer with Cu Kα1 radiation, at an operating voltage and current of 40 kV and 40 mA, respectively, a scanning range of 10° to 80° (2θ), and scanning step setting of 10°/min. The resulting XRD patterns were analyzed using Jade 6.5 software.

#### 2.3.2. Leaching toxicity test.

The raw materials and residues collected after the compressive strength test were ground, and the particles smaller than 100-mesh were selected for the heavy metal leaching test. In this study, the testing steps of “Solid Waste Leaching Toxicity Leaching Method Horizontal Oscillation Method (HJ 557-2010)” [21] and the testing method of “Hazardous Waste Identification Standard Leaching Toxicity Identification (GB5085.3-2007)” [22] were adopted, and the test methods were briefly described as follows. A sample of 100 g (dry weight basis) was taken and placed in a 2-L extraction flask. Based on the moisture content of the sample, the volume of extractant was calculated at a liquid-solid ratio of 10:1 (L/kg), and pure water was added. The bottle was tightly capped and fixed vertically on a horizontal oscillation device, and the oscillation frequency was adjusted to 110 ± 10 times/min and the amplitude to 40 mm. The extraction flasks were removed after 8 hours of oscillation at room temperature. The extraction bottle was then left to stand for 16 hours. If gas was generated during the oscillation process, it was periodically loosened in a fume hood to release excessive pressure. The leachate was filtered through a membrane installed on the pressure filter and analyzed according to the test method specified in “Hazardous Waste Identification Standard for Leaching Toxicity Identification (GB5085.3-2007)”. The detection of heavy metal ions such as Ba, Cd, Cr, Cu, Hg, Mn, Ni, Pb, and Zn was carried out using ICP-MS. The data were then compared with the indices specified in “Surface Water Environmental Quality Standard (GB3838-2002)” [23].

### 2.4. Statistics and analysis

WPS Office was used to process the experimental data. All experiments were repeated in triplicate, and the means ± standard deviations are presented as error bars. Statistical analysis was carried out using WPS Office statistical software and means were compared using the LSD method and one-way ANOVA. *P* < 0.05 was considered to indicate statistical significance. Origin 2018 software was used for data visualization and further analysis.

## 3. Results and analysis

### 3.1. Effect of preparation conditions on RFGP properties

#### 3.1.1. Sodium silicate modulus.

Sodium silicate is a soluble alkali metal silicate material composed of alkali metal oxides and silica. Sodium silicate modulus is an important parameter, which generally refers to the ratio of the amounts of silica to alkali metal oxides (sodium oxide or potassium oxide) in sodium silicate (Na_2_O·nSiO_2_) or potassium silicate (K_2_O·nSiO_2_) [24,25], also known as the mole ratio (or molar ratio). The principle of sodium silicate modulus adjustment is mainly based on changing the relative contents of silica and alkali metal oxides.

The modulus of sodium silicate directly reflects the proportional relationship between silica and alkali metal oxides in its composition [26]. Increasing the silica content or decreasing the alkali metal oxide content increases the modulus of the sodium silicate, and vice versa.

Conditioning methods include chemical methods and mixing methods. Chemically, the modulus can be increased by introducing silica-containing compounds to raise the silica content. Alternatively, acidic substances such as hydrochloric acid and ammonium chloride can be added to neutralize some of the sodium oxides, resulting in a relative increase in the modulus. Conversely, the modulus can be decreased by adding alkaline substances such as sodium hydroxide to increase the content of sodium oxide in the sodium silicate [27,28]. The mixing methods can be used to mix and formulate two different moduli of sodium silicate to obtain the desired modulus of sodium silicate. However, this approach is less commonly used in practice because the performance of the mixed sodium silicate may not be as stable as that of a single-modulus sodium silicate [29]. Therefore, the chemical method was employed in this experiment to reduce the modulus. When sodium hydroxide is added to sodium silicate, the silicate ions in the solution react with silica to alter the silicate structure and release water molecules. This reaction leads to an increase in the sodium oxide content of the sodium silicate, which results in a decrease in the modulus. This chemical reaction can be expressed as:


$${\text{Na}}_{\text{2}}\text{O n}{\text{SiO}}_{\text{2}}+\text{2NaOH}\to {\text{Na}}_{\text{2}}\text{Si}{\text{O}}_{\text{3}}+(\text{n}-\text{1}){\text{SiO}}_{\text{2}}+{\text{H}}_{\text{2}}\text{O}$$ (1)

$${\text{SiO}}_{\text{2}}+\text{2NaOH}\to {\text{Na}}_{\text{2}}\text{Si}{\text{O}}_{\text{3}}+{\text{H}}_{\text{2}}\text{O}$$ (2)

Based on the moduli before and after adjustment of the sodium silicate adjustment, the amount of sodium hydroxide added to the reaction, *Kj* (mol), can be calculated with a conversion factor of 1.29 for NaOH, as shown in the following equation [30]:


$$K_{j}=(\text{Mg}-\text{Md})/(\text{Md}\times \text{P})\times \text{Kc}\times \text{1}.\text{29}$$ (3)

Where *Kj* represents the mass of NaOH added (g); Mg is the modulus of high-modulus sodium silicate; Md is the modulus of low-modulus sodium silicate; Kc is the Na_2_O content in high-modulus sodium silicate (g); and P is the NaOH purity (%).

**Fig 2:**
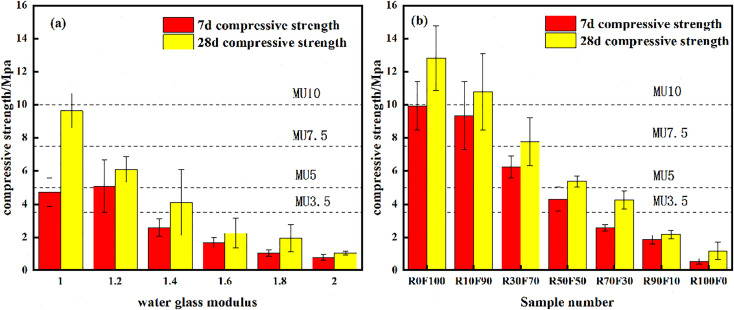
Compressive strength of different sodium silicate moduli (a) and different red mud contents (b).

The effect of alkali activator solution modulus (molar ratio of SiO_2_/Na_2_O) on the compressive strength of RFGP is shown in Fig 2(a). The compressive strength values were compared with the limits specified in the standard “Concrete Plain and Decorative Bricks (NY/T 671-2003)”. From Fig 2(a), it can be seen that the 7-day compressive strength of RFGP initially increases and then decreases with increasing sodium silicate modulus. When the modulus was increased from 1.0 to 1.2, the 7-day compressive strength was increased from 4.7 to 5.1 MPa. When the modulus of sodium silicate was increased from 1.2 to 2.0, the 7-day compressive strength decreased to 0.7 MPa. The decrease in the compressive strength of the specimens was due to the increase in the modulus of the sodium silicate leading to a reduction in the content of Na_2_O and a decrease in the alkalinity, making the geopolymerization reaction incomplete. However, regardless of the alkalinity, the activity of the aluminum silicate crystals in the red mud was too low, resulting in their inability to dissolve completely in the presence of alkali activation [31]. Consequently, the increase in compressive strength was relatively low across all moduli tested, with only a marginal increase observed between moduli of 1.0 and 1.2 [32,33].

The 28-day compressive strength of the geopolymers exhibited a large increase compared to the 7-day strength. As the sodium silicate modulus increased, the compressive strength decreased. When the sodium silicate modulus was 1.0, the 28-day compressive strength reached 9.6 MPa, which exceeded the requirement of MU7.5 in the standard limit of NY/T 671–2003. When the sodium silicate modulus was higher than 1.6, the 28-day compressive strength was even lower than the minimum limit of MU3.5 for non-load-bearing bricks. This may be due to the fact that a large number of oligomeric cyclic polysilicate substances are generated by the excessive concentration of dissolved silica in the hydration reaction process when the sodium silicate modulus is too high [34]. These substances are not conducive to the polymerization between the free alumina-oxygen ligands and the silica-oxygen ligands in the system to generate polyaluminosilicate gel products. Furthermore, they hinder water mobility within the system, adversely affecting the hydration reaction and ultimately reducing the geopolymer strength [35].

Furthermore, comprehensive statistical analysis was conducted using Origin to rigorously evaluate the effects of different treatment conditions on the experimental results.

#### 3.1.2. Raw material proportion.

The effect of raw material proportion on the compressive strength of RFGP is shown in Fig 2(b). With the increase of red mud content, the compressive strength was also reduced. When the red mud content increased from 0% to 30%, the 7-day compressive strength was reduced from 9.9 MPa to 6.2 MPa, and the 28-day compressive strength was reduced from 12.8 MPa to 7.7 MPa, which all met the requirement of MU5 of the non-load-bearing bricks of NY/T 671–2003. At 100% red mud content, the 7-day and 28-day compressive strengths were 1.5 MPa and 1.1 MPa. From the changes in compressive strength at 7-day and 28-day curing ages, it can be seen that the compressive strength increased rapidly in the early curing stage. This phenomenon indicates that the geopolymerization reaction exhibited early strength characteristics. This is because the active aluminosilicate material in fly ash reacted sufficiently in a short time in the alkaline environment to form gels [36].

Additionally, as the red mud content increased from 0% to 10% and subsequently to 30%, the 28-day compressive strength demonstrated a statistically significant decline of 15.8% and 39.3%, respectively (*p*-values < 0.05). These findings substantiate that red mud content also plays a significant role in modulating long-term compressive strength performance. The compressive strength of RFGP decreased rapidly after the addition of more than 30% of red mud. This phenomenon was mainly due to the low SiO_2_ activity in the excess red mud, which hindered the aluminosilicate reaction of the fly ash in the slurry and was not conducive to the formation of a three-dimensional network structure, which in turn reduced the strength of the geopolymer [37]. In addition, due to the small particle size of fly ash, with an average particle size of 500 mesh or less, the particles had a large specific surface area, which can accelerate the reaction rate of the system and fill the space with a smaller size effect to make the system more compact and improve the strength of the geopolymer.

#### 3.1.3. Element ratio proportion.

**Fig 3:**
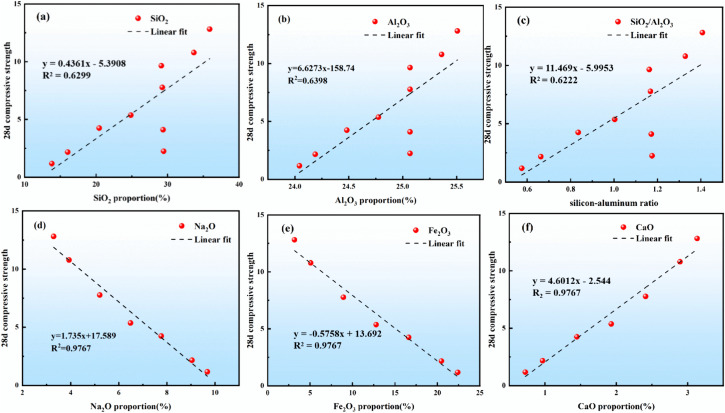
The 28-day compressive strength of various elemental ratios.

As shown in Fig 3, to quantitatively dissect the synergistic influence of silica (SiO₂) and alumina (Al₂O₃) contents—and their ratio—on compressive strength, multivariate linear regression was performed on 28-day strength data obtained from pastes with varying mix proportions. The coefficients of determination (R^2^) for SiO₂ and Al₂O₃ were 0.6299 and 0.9767, respectively, confirming that both oxides contribute significantly to strength development. More importantly, the oxides do not act independently: as the SiO₂/Al₂O₃ ratio increased (R^2^ = 0.6222), strength rose monotonically, demonstrating that the silica-to-alumina ratio functions as a critical interaction parameter that governs the densification of the gel network. This behaviour is attributed to the preferential formation of Si–O–Si and Si–O–Al linkages at high silica-to-alumina ratios, which suppresses the generation of unstable Al–O–Al bridges and thereby stabilises the amorphous three-dimensional framework [38].

Further scrutiny revealed that calcium oxide (CaO) and ferric oxide (Fe₂O₃) are also highly correlated with strength (R^2^ = 0.9767 for each), but their effects are distinctly non-linear and synergistic. CaO refines the pore structure by precipitating C-A-S-H gels, whereas a moderate addition of Fe₂O₃ enhances network cross-linking via Fe–O–Si/Al bonds. However, once Fe₂O₃ exceeds a threshold concentration, its beneficial role reverses: poorly reactive iron oxides form, impeding the geopolymerization reaction and causing strength loss. Consequently, CaO and Fe₂O₃ exhibit a dose-dependent switch from synergy to competition [39].

In contrast, sodium oxide (Na₂O) exerts a pronounced antagonistic effect. Increasing Na_2_O systematically reduces 28-day compressive strength. The underlying mechanism involves the compression of the electrical double layer by elevated Na^+^ concentrations, which weakens the electrostatic repulsion between AlO_4_^-^ and Si-O^-^ units. The resulting Al-O-Si linkages become vulnerable to nucleophilic attack by OH^-^, yielding low-molecular-weight oligomers and terminal hydroxyl groups (Si–OH, Al–OH) that hinder the formation of a dense three-dimensional gel. Simultaneously, the high-alkali environment fosters the crystallisation of sodium-rich zeolites (e.g., natrolite and analcime), generating micro-cracks and interfacial stresses that further degrade macroscopic strength. Notably, the detrimental influence of Na₂O transcends single-factor boundaries: even when the SiO_2_/Al_2_O_3_ ratio is optimised, high Na^+^ levels can offset the silica–alumina synergy by suppressing condensation reactions [40].

Collectively, the strength evolution of geopolymer binders is not a simple superposition of individual oxide contributions; rather, it is governed by multifactor interactions, non-linear responses, and threshold effects. SiO_2_ and Al_2_O_3_ constitute the primary network backbone, and their ratio serves as the key order parameter that tailors the gel architecture. CaO and Fe_2_O_3_ act synergistically within a narrow dosage window, yet Fe_2_O_3_ excess triggers performance reversal. Na_2_O functions as a systematic inhibitor whose accumulation broadly disrupts polycondensation and product stability [40].

### 3.2. Characterization of RFG

#### 3.2.1. SEM.

**Fig 4:**
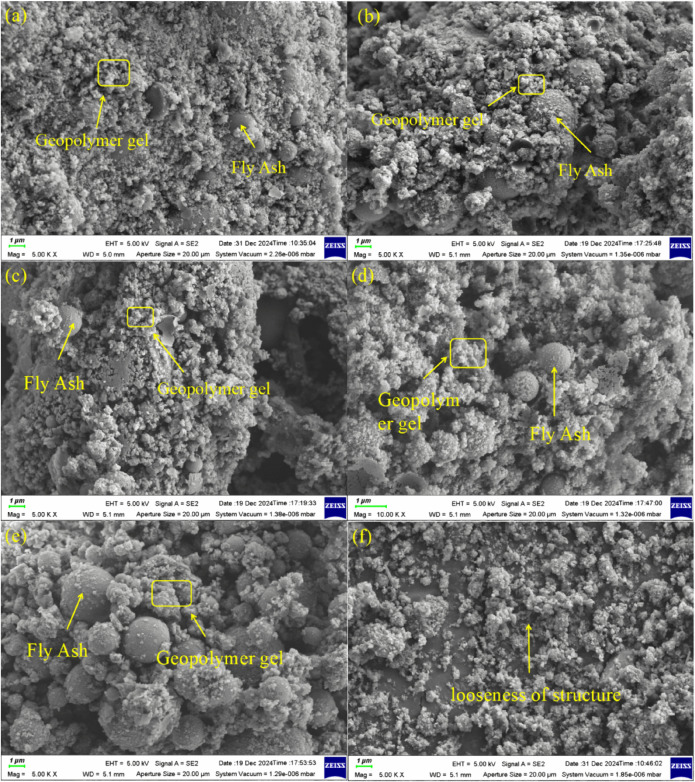
The SEM images of RFG samples at 28 days (R00F100 (a), R30F70 (b), R70F30 (c), R30F70-1.2 (d), R30F70-1.8 (e) and R100F0 (f)).

The micrographs of the geopolymers with different treatments at 28 days are shown in Fig 4. With increasing red mud content (from R0F100 to R100F0), the microstructure became progressively looser. The internal pores were filled with minerals from reactions and from raw materials, so the number of pores and defects was reduced, and the microstructure was more continuous. Compared with R30F70, R70F30, the higher the red mud content, the looser the microstructure. R70F30 had less gel and a more discontinuous structure. R30F70 geopolymers had higher density, less unreacted particles, and tighter structures. As shown in Fig 4(f), the geopolymerization reaction did not occur to generate gel, and the particles were loose without forming a reticulated three-dimensional structure. Therefore, the 28-day compressive strength of R70F30 was only 1.175MPa. This indicates that the activity of RM was low under the same conditions, so when the dosage of the RM increased, the compressive strength decreased. Between R30F70-1.2 and R30F70-1.8, R30F70-1.2 had a more compact microstructure. It had fewer unreacted FA particles and pores. The R30F70-1.8 had larger FA particles and a more dispersed structure. This showed that the lower the sodium silicate modulus, the greater the alkalinity, and the more complete geopolymerization. High alkalinity promoted the formation of N-A-S-H gel, which incorporated Na+ into the aluminosilicate framework. As a result, a strong three-dimensional reticulated gel structure was formed, whose internal pores were filled with minerals produced by the reaction and minerals from the raw material [41].

#### 3.2.2. TG-DTG.

**Fig 5:**
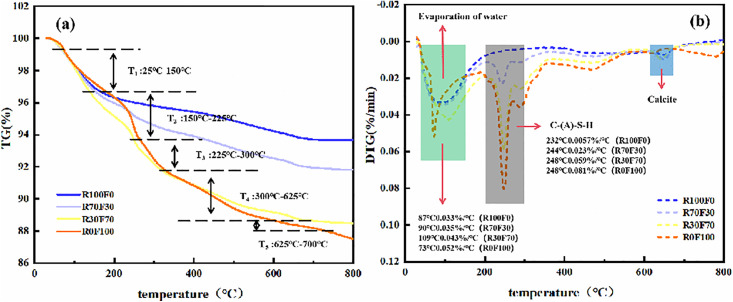
The TG (a) and DTG (b) curves of RFGP geopolymer.

The TG-DTG plots of geopolymers with different treatments are shown in Fig 5. During the heating process, the gel products in the geopolymer were gradually dehydrated and the mass of the specimens decreased continuously. Differential thermogravimetric (DTG) curves showed that the mass of the geopolymers decreased significantly at temperatures ranging from 50°C to 150°C and from 225°C to 300°C. The peak in mass loss before 150°C could be attributed to the evaporation of free water [42]. From 225°C to 300°C, all samples showed significant mass loss peaks, which were mainly related to the dehydration of C-(A)-S-H and N-A-S-H gels [43]. Both the temperature range and the peak magnitude agree closely with the benchmark values reported by Myers et al. [44] for pure C-(A)-S-H (peak at 270°C, mass loss 3.5%), confirming that this peak can serve as an indicator of gel content in the geopolymer. Focusing on the peak between 225°C and 300°C and comparing the differences, it was observed that the mass loss peak increased with higher red mud content. This finding was consistent with the result of XRD analysis and explained the trend of compressive strength change. Comparing the differences in this peak, it was found that as the RM content increased from 10% to 30%, the decrease in mass loss peak area was less pronounced. However, when the RM content exceeded 30%, the change in the mass loss peak of the DTG curve was significant, which indicates that a small amount of RM had little effect on the gel generation, but a higher percentage of RM might affect the geopolymer gel generation. In addition, a new mass-loss peak appeared at 625–700°C, originated from calcite decomposition and the release of CO_2_, corroborating the presence of carbonate impurities [45].

#### 3.2.3. FTIR.

**Fig 6:**
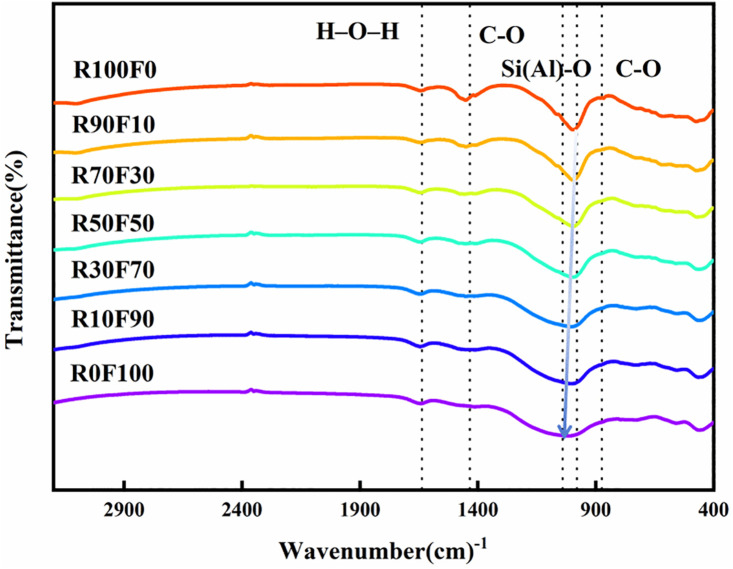
The FT-IR pattern of RFGP geopolymer.

The FT-IR plots of the geopolymers with different treatments are shown in Fig 6. In all raw material samples and geopolymer samples, an absorption band located at 980 cm-1 can be observed, corresponding to asymmetric stretching motions of Si-O or Al-O. This band indicates the formation of C-(A)-S-H gels. Furthermore, the absorption bands located at 1434 cm^-1^ and 874 cm^-1^ correspond to stretching vibrations of C-O, and the absorption band at 1627 cm^-1^ corresponds to bending vibrations of H-O-H [46].

S2 Fig shows the FT-IR characterization of the raw materials red mud and fly ash. Fig 6 shows the FT-IR characterization of RFGP. The FT-IR spectra of the RFGP geopolymers changed compared to those of the raw materials, indicating that geopolymerization occurred during the alkali activation process. Variations in the positions and intensities of the Si(Al)–O bands characterize the formation of geopolymer gels. As the red mud content decreased, the infrared spectra changed: the main band shifted from 980 cm^-1^ to 1040 cm^-1^ (a 60 cm^-1^ shift to higher wavenumbers) and the amorphous asymmetric band became less pronounced. This was attributed to reduced geopolymerization efficiency at higher red-mud contents [47]; consequently, the 28-day compressive strength increased with decreasing red-mud content.

#### 3.2.4. XRD.

**Fig 7:**
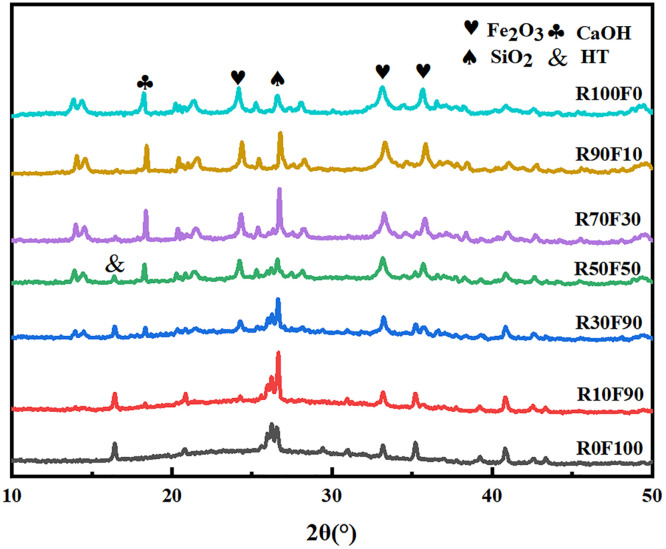
The XRD pattern of RFGP geopolymer.

The samples with different red mud contents were analyzed by XRD, and the results were shown in Fig 7. Five mineral phases were present in different samples at a curing age of 28 days: hematite [Fe_2_O_3_], quartz [SiO_2_], calcium hydroxide [Ca(OH)_2_], calcium oxide [CaO], and hydrotalcite (HT) [Mg_6_Al_2_(OH)_16_](CO_3_)⋅12H_2_O]. The generation of HT indicated a geopolymer gel that distinguishes the alkali-activated geopolymer from the unreacted material [48]. Hematite (Fe_2_O_3_) is a mineral phase derived from RM and did not participate in the geopolymerization reaction. A broad amorphous hump can be observed at 25° to 35° for R0F100, R10F90 and R30F70. This halo corresponded to a mixed aluminosilicate gel consisting of N-A-S-H, C-A-S-H and C-(N)-A-S-H, whose three-dimensional silicate/aluminate framework was formed via condensation of [SiO_4_]^4-^ and [AlO_4_]^5-^ tetrahedra under the catalysis of OH- ions. The intensity of the quartz peaks decreased systematically with increasing red mud content, indicating that the crystalline SiO_2_ is partially depolymerised into soluble monomeric [SiO_4_] units [49]. The crystal diffraction peaks... decreased with decreasing red mud content, reflecting the enhanced dry basis activity of the geopolymer. These minerals were partially dissolved into silica-aluminate by alkali activation, and geopolymer gels were gradually generated [50].

With increasing red-mud addition, the XRD patterns of samples containing more than 70 wt % red mud became almost identical to those of the raw red mud, and the broad hump at 25–35° 2θ became barely visible. This indicates that red-mud contents above 70% hinder effective geopolymerization. The crystalline phases abundant in red mud are only partially decomposed under alkaline activation, yielding an insufficient volume of gel. Large inter-granular voids remained, and the residual compressive strength was therefore controlled by weak van der Waals interactions between unreacted red-mud particles rather than by the strong covalent Si-O-Al framework. Macroscopically, this is reflected in a sharp decline in 28-day compressive strength once the red-mud fraction exceeds 70% [51,52]. Ultimately, too little gel forms to consolidate the system, and excessive red-mud addition reduces reactivity and suppresses geopolymerization.

### 3.3. Stabilization and solidification of heavy metals

**Fig 8:**
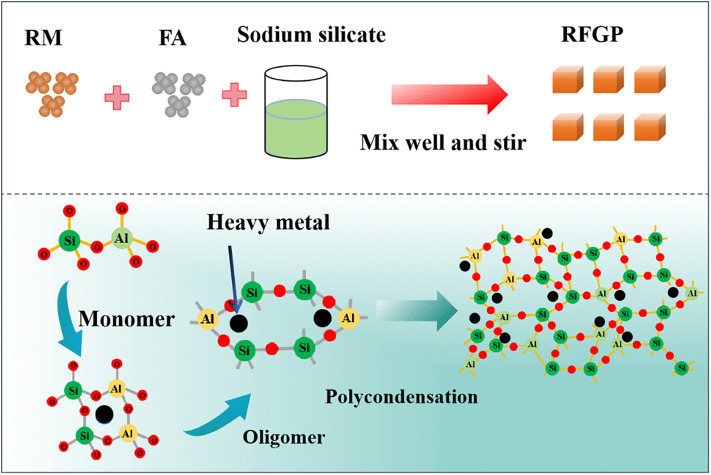
Schematic of RFGP geopolymer heavy metal solidification mechanism.

Some red mud may contain heavy metal elements that are hazardous waste. Its storage can cause serious pollution of air, soil, and water, which imposes a significant burden on the environment. Therefore, the utilization and disposal of hazardous solid waste have attracted the attention of both academia and industry. According to the statement of the United States Environmental Protection Agency (USEPA), solidification/stabilization technology is one of the most effective methods for the disposal of wastes containing hazardous heavy metals [53,54]. Geopolymer is one of the commonly used materials for curing/stabilizing heavy metals [55]. It has the advantages of high solidification/stabilization efficiency, high solid-waste utilization rate, simple process, and environmental friendliness [56,57]. The RFGP heavy-metal solidification mechanism is illustrated in Fig 8.

The results of heavy metal leaching from red mud, fly ash, and R30F70 geopolymer are shown in Table 3, and the testing procedure was adopted from the “Solid waste-Extraction procedure for leaching toxicity- Horizontal Vibration Method (HJ 57-2010)”. The results indicated that the leaching amount of Ba and Hg in red mud reached 0.0166 mg/L and 0.0049 mg/L, respectively, and the leaching amount of Hg in fly ash was 0.0021 mg/L. The leaching concentration of Ba and Hg in raw materials did not comply with the standards of Class IV and Class V water in the “Environmental quality standards for surface water (GB 3838-2002) ”, but neither of them exceeded the concentration limit value of the “Identification standards for hazardous wastes-Identification for extraction toxicity (GB 5085.3-2007) ”. The leaching results of the R30F70 geopolymer showed that only the Ba leaching amount reached 0.0075 mg/L, which did not meet the standard of GB 3838−2002 Class IV water but did not exceed the concentration limit in GB 5085.3−2007. In addition, the leaching results of R30F70 geopolymer showed that most of the heavy metals were reduced to different degrees compared with the raw material. For example, the leaching amount of Hg, which had exceeded the standard, was reduced to 0.0004 mg/L. The concentrations of heavy metal ions in the samples were greatly reduced compared with those in the raw material before the reaction, which basically complied with the standard. This may be due to the fact that the geopolymer reacted to produce geopolymerization products (e.g., C-(A)-S-H gel) to promote the solidification/stabilization of heavy metals such as Hg [58,59].

**Table 3:** Leaching toxicity of RFGP geopolymer.

Samples		Ba	Cd	Cr	Cu	Hg	Mn	Ni	Pb	Zn
RM		0.0166	0.0006	0.0653	0.0041	0.0049	0.0141	0.0113	0.0092	0.0721
FA		0.0035	0.0001	0.0466	0.0007	0.0021	0.001	0.0103	0.0182	0.0599
R30F70		0.0075	0.0004	0.2205	0.0008	0.0004	0.003	0.0059	0.0178	0.0423
GB5085.3−2007		100	1	15	100	0.1	/	5	5	100
GB3838−2002	Ⅲ	0.005	0.005	/	1	0.0001	/	/	0.05	1
	Ⅳ	0.005	0.005	/	1	0.001	/	/	0.05	2
	Ⅴ	0.01	0.01	/	1	0.001	/	/	0.1	20

## 4. Conclusion

This paper proposes a method for preparing red mud-fly ash geopolymers using red mud and fly ash as raw materials and sodium silicate and sodium hydroxide as alkali activators. Building on this basis, the effects of raw material ratios and alkali activator modulus on the properties of geopolymer were investigated; the optimal ratios were determined, and the strength formation mechanism of geopolymer and the geopolymerization reaction mechanism were studied. At the same time, the heavy metal ion concentrations of the leachates were tested. The immobilization effect of the geopolymer on heavy metal ions was clarified. The main conclusions are as follows.

(1) **Different Starting Materials Affect Compressive Strength**

With the increase of sodium silicate modulus, the compressive strength showed a decreasing trend, and the 7-day and 28-day compressive strengths were 5.1 MPa and 6.1 MPa, respectively, at the sodium silicate modulus of 1.2. The decreasing trend accelerated after the addition of more than 30% of red mud. At red mud addition levels of 10% and 30%, the 28-day compressive strengths were 10.78 MPa and 7.78 MPa, respectively. These results demonstrate that higher red mud content led to lower strength, with a more rapid decrease observed beyond 30% replacement.

(2) **Effect of Elemental Proportion on Compressive Strength Development.**

With the increase of red mud addition, the Si/Al ratio and CaO of geopolymer decreased. Meanwhile, the Na_2_O and Fe_2_O_3_ percentages increased, and the 28-day compressive strength showed a decreasing trend. This study showed that higher Si/Al ratios and CaO contents promote the formation of geopolymer gel phases, enhancing structural density and mechanical performance. Furthermore, excessive Na_2_O and Fe_2_O_3_ hindered the polymerization reaction and reduced the compressive strength.

(3) **Stabilization and Solidification of Heavy Metals.**

The immobilization and stabilization of heavy metals were accomplished by the red mud-based geopolymer through physical encapsulation, adsorption, and chemical bonding of metal ions in the reaction products. The concentration of heavy metals in the leaching solution of the specimens did not exceed the limits specified in GB 3838−2002, indicating that this material has strong potential for heavy metal pollution control.

## Supporting information

S1 Fig The XRD pattern of red mud and fly ash. (TIF)

S2 Fig The IR pattern of red mud And fly ash. (TIF)

S1 Table Compressive Strength of Water Glass with Different Moduli (Fig 2(a)). (DOCX)

S2 Table Compressive strength of different raw material ratios (Fig 2(b)). (DOCX)

S3 Table The proportion of each element and the 28d compressive strength in different red mud mixing experiments (Fig 3). (DOCX)
