# Supplementary material for: Synergistic effects of high-temperature curing and elemental conditioning on red mud-based geopolymer: Compressive strength and immobilization
Source: PLoS One. 2026 Apr 20;21(4):e0343975. doi: 10.1371/journal.pone.0343975 (PMC13094981; doi:10.1371/journal.pone.0343975)
Supplement: S1 Table — (DOCX) [file pone.0343975.s003.docx]

**Table S1** Compressive Strength of Water Glass with Different Moduli **(Fig 2(a))**

| number | 7d compressive strength/MPa | 7d compressive strength/MPa | 7d compressive strength/MPa | 28d compressive strength/MPa | 28d compressive strength/MPa | 7d means | 7d standard deviation/MPa | 28d  means | 28dstandard deviation/MPa |
| --- | --- | --- | --- | --- | --- | --- | --- | --- | --- |
| R30F70-1.0 | 4.10625 | 5.33125 | 7.225 | 10.375 | 8.91875 | 4.71875 | 0.86621 | 9.64687 | 1.02972 |
| R30F70-1.4 | 3.9625 | 6.2125 | 5.2625 | 5.55625 | 6.64375 | 5.0875 | 1.59099 | 6.1 | 0.76898 |
| R30F70-1.6 | 2 | 2.7375 | 3 | 2.7 | 5.50625 | 2.57917 | 0.51846 | 4.10313 | 1.98432 |
| R30F70-1.8 | 1.4625 | 1.895 | 1.66875 | 1.6 | 2.8875 | 1.67875 | 0.30582 | 2.24375 | 0.9104 |
| R30F70-2.0 | 0.89375 | 1.1875 | 1.2125 | 1.3625 | 2.53813 | 1.04062 | 0.20771 | 1.95031 | 0.83129 |
| R30F70-1.0 | 0.90625 | 0.84375 | 0.6 | 1.11875 | 0.96875 | 0.78333 | 0.16182 | 1.04375 | 0.10607 |
