# Supplementary material for: Synergistic effects of high-temperature curing and elemental conditioning on red mud-based geopolymer: Compressive strength and immobilization
Source: PLoS One. 2026 Apr 20;21(4):e0343975. doi: 10.1371/journal.pone.0343975 (PMC13094981; doi:10.1371/journal.pone.0343975)
Supplement: S2 Table — (DOCX) [file pone.0343975.s004.docx]

**Table S2** Compressive strength of different raw material ratios **(Fig 2(b))**

| number | 7d compressive strength/MPa | 7d compressive strength/MPa | 7d compressive strength/MPa | 28d compressive strength/Mpa | 28d compressive strength/Mpa | 28d compressive strength/Mpa | 7d means/Mpa | 7d standard deviation/Mpa | 28d means/Mpa | 28dstandard deviation/Mpa |
| --- | --- | --- | --- | --- | --- | --- | --- | --- | --- | --- |
| R30F70-1.0 | 4.10625 | 5.33125 | 7.225 | 10.85631 | 12.815 | 14.77369 | 9.92667 | 1.45772 | 12.815 | 1.95869 |
| R30F70-1.4 | 3.9625 | 6.2125 | 5.2625 | 8.47102 | 10.78667 | 13.10232 | 9.34333 | 2.06083 | 10.78667 | 2.31565 |
| R30F70-1.6 | 2 | 2.7375 | 3 | 6.32543 | 7.775 | 9.22457 | 6.25 | 0.65054 | 7.775 | 1.44957 |
| R30F70-1.8 | 1.4625 | 1.895 | 1.66875 | 5.02852 | 5.375 | 5.72148 | 4.295 | 0.71418 | 5.375 | 0.34648 |
| R30F70-2.0 | 0.89375 | 1.1875 | 1.2125 | 3.71166 | 4.24667 | 4.78168 | 2.565 | 0.20506 | 4.24667 | 0.53501 |
| R30F70-1.0 | 0.90625 | 0.84375 | 0.6 | 1.91009 | 2.16667 | 2.42325 | 1.855 | 0.26163 | 2.16667 | 0.25658 |
