# Supplementary material for: Synergistic effects of high-temperature curing and elemental conditioning on red mud-based geopolymer: Compressive strength and immobilization
Source: PLoS One. 2026 Apr 20;21(4):e0343975. doi: 10.1371/journal.pone.0343975 (PMC13094981; doi:10.1371/journal.pone.0343975)
Supplement: S3 Table — (DOCX) [file pone.0343975.s005.docx]

**Table S3** The proportion of each element and the 28d compressive strength in different red mud mixing experiments **(Fig 3)**

| number | SiO_2_(%) | Al_2_O_3_(%) | SiO_2_/Al_2_O_3_ | Na_2_O(%) | Fe_2_O_3_(%) | CaO(%) | 28d compressive strength/MPa |
| --- | --- | --- | --- | --- | --- | --- | --- |
| R0F100 | 12.351 | 9.801 | 1.2592 | 2.087 | 3.155 | 3.134 | 12.815 |
| R10F90 | 11.615 | 9.760 | 1.1842 | 2.725 | 5.079 | 2.893 | 10.787 |
| R30F70 | 10.145 | 9.662 | 1.4995 | 4.003 | 8.929 | 2.411 | 7.775 |
| R50F50 | 8.674 | 9.565 | 0.9067 | 5.280 | 12.778 | 1.929 | 5.375 |
| R70F30 | 7.203 | 9.467 | 0.7608 | 6.558 | 16.627 | 1.448 | 4.247 |
| R90F10 | 5.732 | 9.369 | 0.6118 | 7.835 | 20.478 | 0.966 | 2.167 |
| R100F0 | 4.996 | 9.320 | 0.5361 | 8.747 | 22.401 | 0.725 | 1.175 |
